# Management of Complex Small Bowel Obstructions of Various Etiologies: A Case Series and Literature Review

**DOI:** 10.7759/cureus.69487

**Published:** 2024-09-15

**Authors:** Abshiro H Mayow, Jetvir Singh, Elizabeth Edah, Frederick Tiesenga

**Affiliations:** 1 Department of Medicine, St. George's University, St. George's, GRD; 2 Department of Surgery, Windsor University School of Medicine, Cayon, KNA; 3 Department of General Surgery, All Saints Medical School, Roseau, DMA; 4 Department of General Surgery, West Suburban Medical Center, Chicago, USA

**Keywords:** adenocarcinoma, adhesions, exploratory laparotomy, gastrointestinal obstruction, small bowel obstruction

## Abstract

A small bowel obstruction (SBO) involves partial or complete blockage of the small intestine. Complete high-grade or closed-loop obstructions constitute acute surgical emergencies necessitating urgent intervention to prevent severe complications. Several probable etiologies of SBO have been identified, including malignancy, stool impaction, and inflammatory bowel disease; however, post-surgical adhesion is the most prevalent cause in the developed world. A SBO can be diagnosed clinically after a physical examination, but imaging is needed for surgical assessment and management. Misdiagnosis and inadequate management of SBO can cause several complications, such as intestinal necrosis and perforation. Therefore, when required, rapid diagnosis and surgical intervention are critical to avoiding mortality. Computer tomography (CT) and small bowel follow-through (SBFT) are widely utilized imaging modalities for confirmatory diagnosis of SBO. This case series features three patients who had exploratory laparotomies following extensive clinical evaluations and a radiologically confirmed diagnosis of SBO. All three cases involved numerous surgeries. The patients presented with severe abdominal discomfort and received surgical intervention after a CT scan confirmed the diagnosis and conservative therapy failed. In this case, all patients had different presentations but responded adequately to the same treatment modalities with no postoperative complications.

## Introduction

Small bowel obstruction (SBO) is characterized by partial or complete obstruction of the small intestine, leading to the disruption of the normal flow of intestinal intraluminal contents. It can occur due to genetic or acquired factors. Small bowel obstruction can be further categorized as strangulated or non-strangulated [1]. Patients with a history of abdominal surgeries or trauma are most likely to develop SBO due to intra-abdominal adhesions. Increased intraluminal pressure leads to endothelial tissue remodeling, which induces changes in the epithelial type, allowing it to withstand the greater forces exerted by extraluminal factors such as tumors and adhesions [2]. Moreover, adhesions or thickening of fibrous tissues of the small intestine replace simple columnar epithelial cells and block gastrointestinal absorption, thus interfering with the normal functions of the small intestines [1]. Therefore, patients with SBO typically present with symptoms of severe abdominal pain, vomiting, obstipation, constipation, and abdominal distention [3]. Common causes of SBO include hernias, volvulus, malignancy, inflammatory bowel disease, stool impaction, and foreign objects.

While a physical examination has been traditionally used to diagnose SBO, advanced imaging techniques such as computer tomography (CT) scans and small bowel follow-through (SBFT) are helpful for prompt diagnosis. This can help avoid delays in treatment and reduce the risk of complications. A CT scan of the abdomen is one of the vital imaging modalities. Intravenous (IV) contrast should be used if the patient has normal renal function and is not contraindicated. A non-contrast study may be obtained if the patient has a subnormal renal function [4,5]. A routine laboratory examination should also be conducted to evaluate for bowel ischemia and inflammation. These tests may include a complete blood count (CBC), lactic acid level, metabolic profile (CMP), urine analysis, and coagulation analysis [4]. An increased white blood cell (WBC) count, fever, and a dilated small bowel measuring more than 3 cm indicate a higher probability of bowel compromise.

This series discusses three specific cases of SBO with diverse etiologies, complex medical histories, and varied presentations. Adenocarcinoma, excessive adhesions, and comorbid conditions are associated with a significant risk of recurrent SBO, which is the focus of this case series [6]. This report aims to contribute to the existing literature by describing a practical approach to managing SBOs in patients with complicated medical histories despite various etiologies or factors causing the acute obstruction. To prevent complications and ensure favorable patient outcomes, timely interventions were crucial.

## Case presentation

Case one

**Figure 1:**
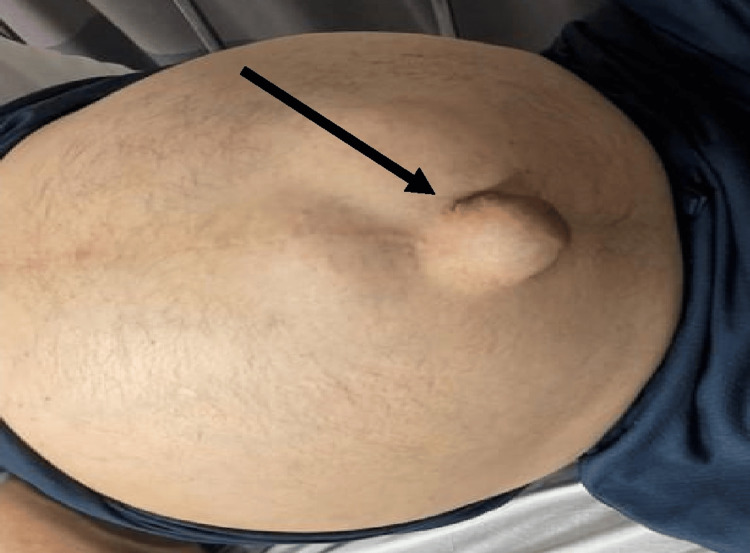
Case one: distended abdomen with protruding umbilical hernia (black arrow)

A 53-year-old male presented to the emergency department with severe and persistent abdominal pain. The patient’s medical history included a perforated duodenal ulcer treated with exploratory laparotomy. Five years ago, the patient underwent a cholecystectomy, and last year, esophagogastroduodenoscopy with banding was performed six times. His physical examination revealed a soft, distended abdomen with diffuse tenderness, a protruding soft umbilical hernia with a 3 cm fascial defect, and caput medusa. Figure 1 shows a distended abdomen with a visible umbilical hernia.

**Figure 2:**
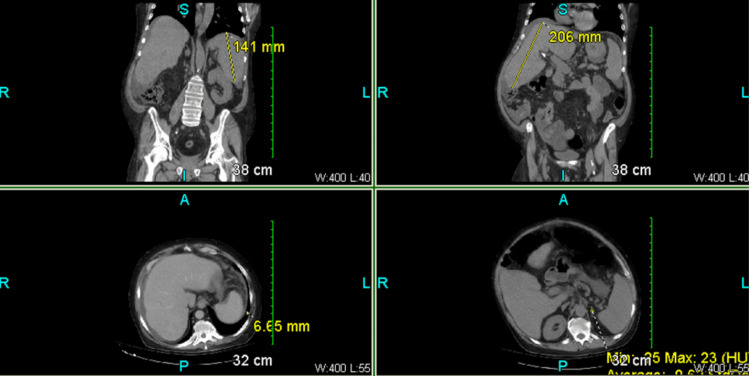
Case one: abdominal CT images showing a 7 mm left lung nodule, cirrhotic liver, ascites, dilated small bowel loops, and decompressed distal bowel

The patient had an elevated blood pressure of 161/89 mmHg and a pulse rate of 97 beats per minute. His laboratory results showed an elevated glucose level of 359 mg/dL (normal range: 70-99 mg/dL) and a low hemoglobin level of 10.7 g/dL (normal range: 13.8 to 17.2 g/dL). Further investigation with abdominal CT images, as shown in Figure 2 below, revealed multiple findings, including a 7 mm nodule at the left lung base, a cirrhotic liver, a small amount of ascites, dilated fluid-filled loops of the small bowel, decompressed distal small bowel with stool present, a transition point in the right lower quadrant, and a small fluid-containing umbilical hernia.

**Figure 3:**
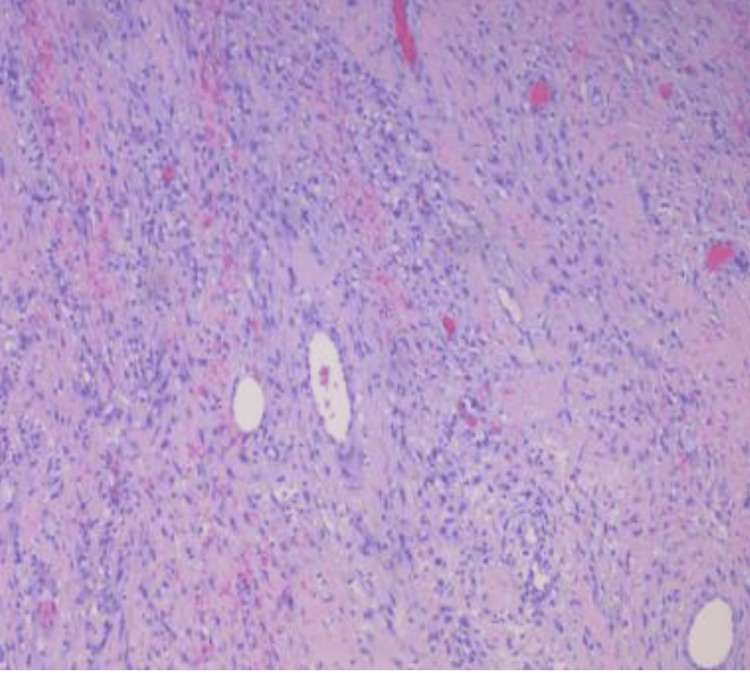
Case one: histopathological analysis demonstrates significant fibrosis, granulation tissue formation, vascular congestion, and reactive alterations, primarily localized to the serosal surface.

The findings indicated a possible acute-on-chronic SBO with a transition point in the right lower quadrant. In addition, liver cirrhosis with ascites and a 7 mm nodule in the left lower lung were also detected. Fecalization of enteric contents was seen in the distal small bowel, which was suggestive of chronic low-grade or partial obstruction. After 24 hours of unresolved obstruction despite nasogastric tube placement, it was determined that a laparotomy and resection of the necrotic bowel were necessary due to the failure of conservative management. Furthermore, the single supine view of the upper abdomen X-ray demonstrated mildly distended small bowel segments. The patient underwent an exploratory laparotomy, small bowel resection, and incisional and ventral hernia repair. Figure 3 shows a photomicrograph of the resected small intestine.

The procedure was successful, and the patient remained stable throughout his hospital stay. On postoperative day (POD) six, the patient was discharged and advised to undergo physiotherapy, a CT scan, and an outpatient follow-up. This patient qualified for CT follow-up for one of two critical reasons: A nodule of 7 mm in size in the left lower lobe constituted the symptomatic liver metastasis, so there has been a recommendation to perform a follow-up CT of the chest in three to six months. There is a need for routine yearly imaging, as the American Society of Colon and Rectal Surgeons recommends for patients who are under treatment for colon or rectal adenocarcinoma yearly imaging, including CT scans of the chest, abdomen, and pelvis because such patients are at risk for developing metastasis. In the current scenario, annual CT scans would be useful in monitoring the tumor regrowth and also preventing the incidences of recurrent bowel obstructions at an early stage, which would call for correction of the situation to prevent any further complications.

Case two

A 59-year-old male presented to the emergency department with a progressive, sudden onset of epigastric abdominal pain radiating to the lower left quadrant. The patient reported associated symptoms of nausea, vomiting, and diarrhea with blood streaks. His medical history included locally advanced rectal adenocarcinoma with hepatic metastases, treated one year prior with surgical excision and end colostomy creation. The patient also received palliative chemotherapy. Additionally, the patient had a history of diabetes mellitus, hypertension, and hyperlipidemia. 

**Figure 4:**
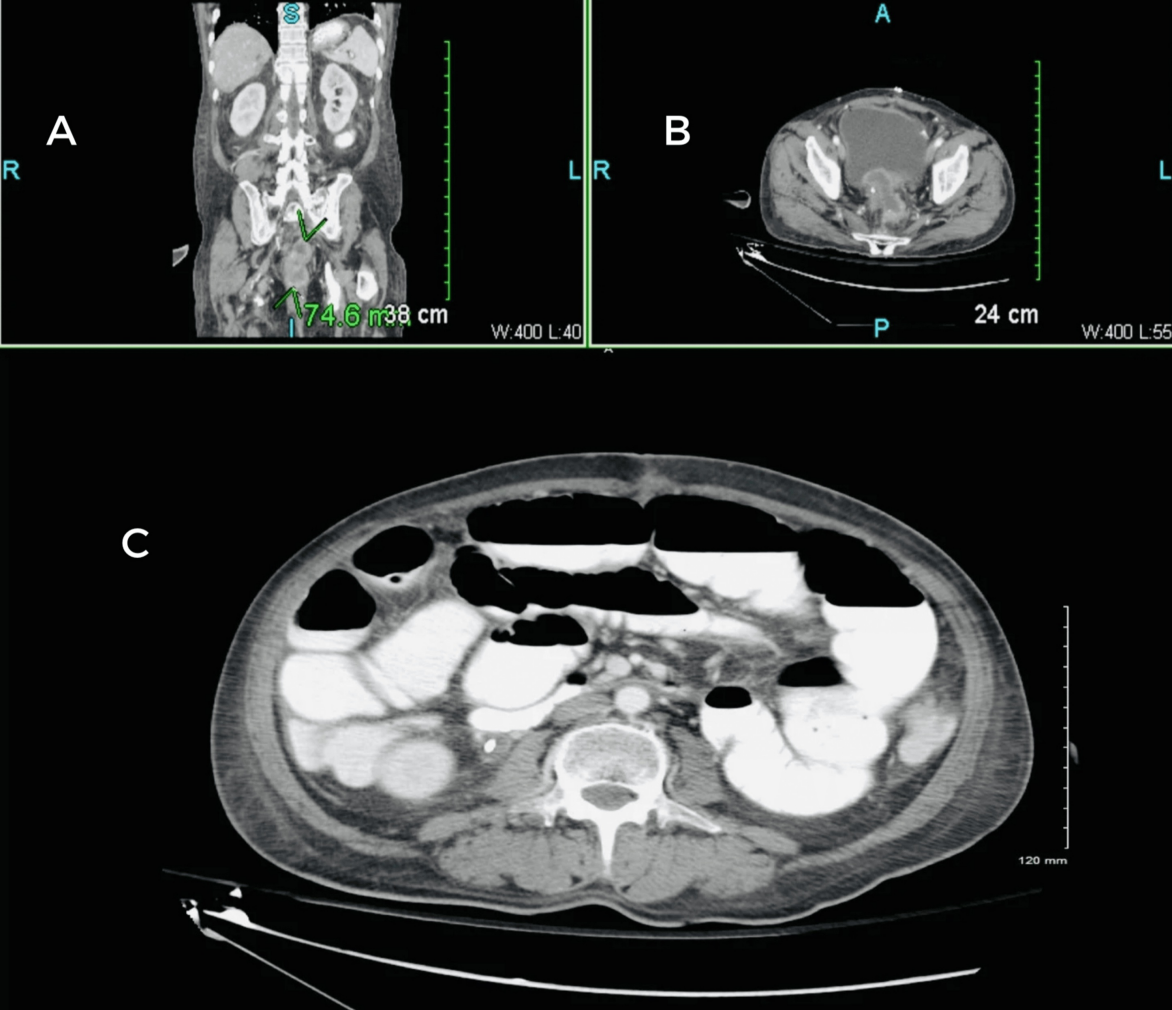
Case two: abdomen CT findings include (A) many metastatic liver lesions of different persuasions; (B) a 7.0 x 4.6 x 7.57 irregular necrotic of the rectosigmoid with necrotic ela area extending bladder wall; (C) moderate distribution of gas and air-fluid levels in the small bowel loops within the abdomen consistent with obstruction.

The physical examination revealed a soft but generalized tenderness in the abdomen, with no signs of peritonitis. Other physical parameters of the patient appeared normal. The complete blood count showed that most parameters were within normal range. The comprehensive metabolic panel (CMP) showed elevated glucose at 252 mg/dL (normal range: 70-99 mg/dL) and alkaline phosphatase at 207 IU/L (normal range: 40-129 IU/L). A CT scan revealed multiple dilated loops of the small bowel and a transition point in the pelvic ileum, adjacent to a dilated segment containing fecal material. Ascites was present, but no free intraperitoneal air was detected. A left anterior abdominal wall colostomy was noted. Moreover, a rectal area mass was found, extending anteriorly and rightward to the right posterior urinary bladder wall. The CT scan demonstrated multiple liver metastases and retained fluid in the distal esophagus. It confirmed the presence of SBO with a defined transition point and no evidence of pneumoperitoneum. A nasogastric tube was placed, with the tip and side port overlying the gastric fundus, and a right ureteral stent was also present. Spinal degenerative changes, left base atelectasis, and a left subclavian central venous port were noted. Figure 4 shows a CT scan of the abdomen and pelvis.

**Figure 5:**
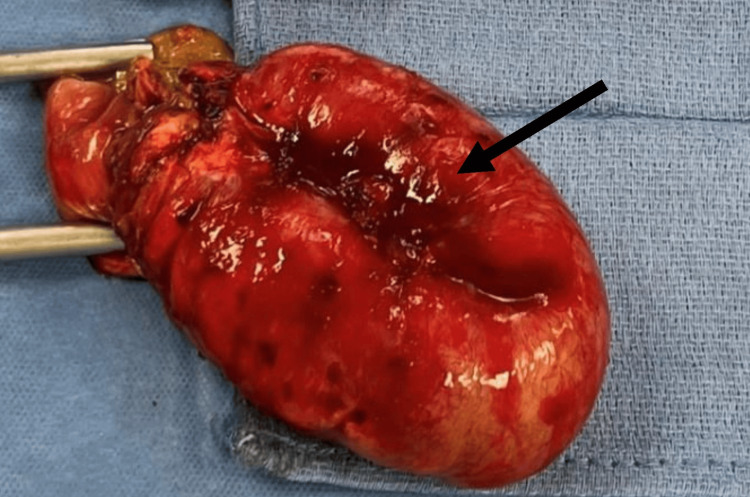
Case two: resected adhesion mass during the procedure

The patient was managed non-operatively with IV fluids for 24 hours without resolution. Small bowel flow through indicated significant obstruction due to a massive tumor. The patient’s WBC had remained elevated. Thus, the patient agreed to the option of having surgery and chose to proceed with it. This case highlights the importance of offering surgical options to patients and not assuming they are not candidates for surgery. Figure 5 shows resected adhesion mass.

**Figure 6:**
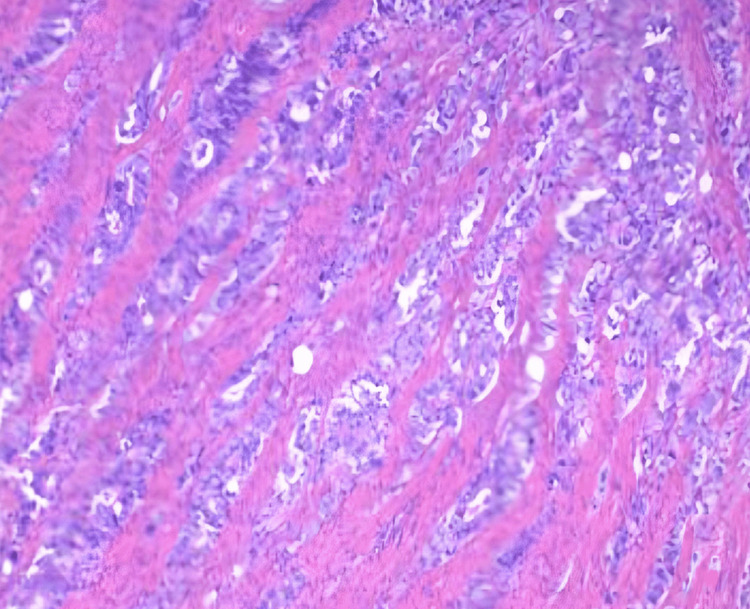
Case two: small bowel tissue showing serosal surface malignant glands

A histopathological examination was performed to investigate the nature of the mass further and guide appropriate next-step management. Figure 6 shows histopathology findings of the resected part of the small intestine.

Immunohistochemistry showed that the glands were positive for CDX2, CK20, and CK7. Post-surgical hospitalization was uneventful. The patient was discharged, and outpatient follow-up was recommended.

Case three

A 39-year-old male presented to the emergency department complaining of severe right-sided abdominal pain that felt like a sharp pressure. His surgical history included exploratory laparotomy for a gunshot wound 18 years ago, SBO repair 17 years ago, and inguinal hernia repair eight months ago. Although he denied current symptoms, he reported experiencing multiple episodes of bowel obstruction that usually resolved spontaneously at home. However, he also required hospitalization and nasogastric tube placement on several occasions.

**Figure 7:**
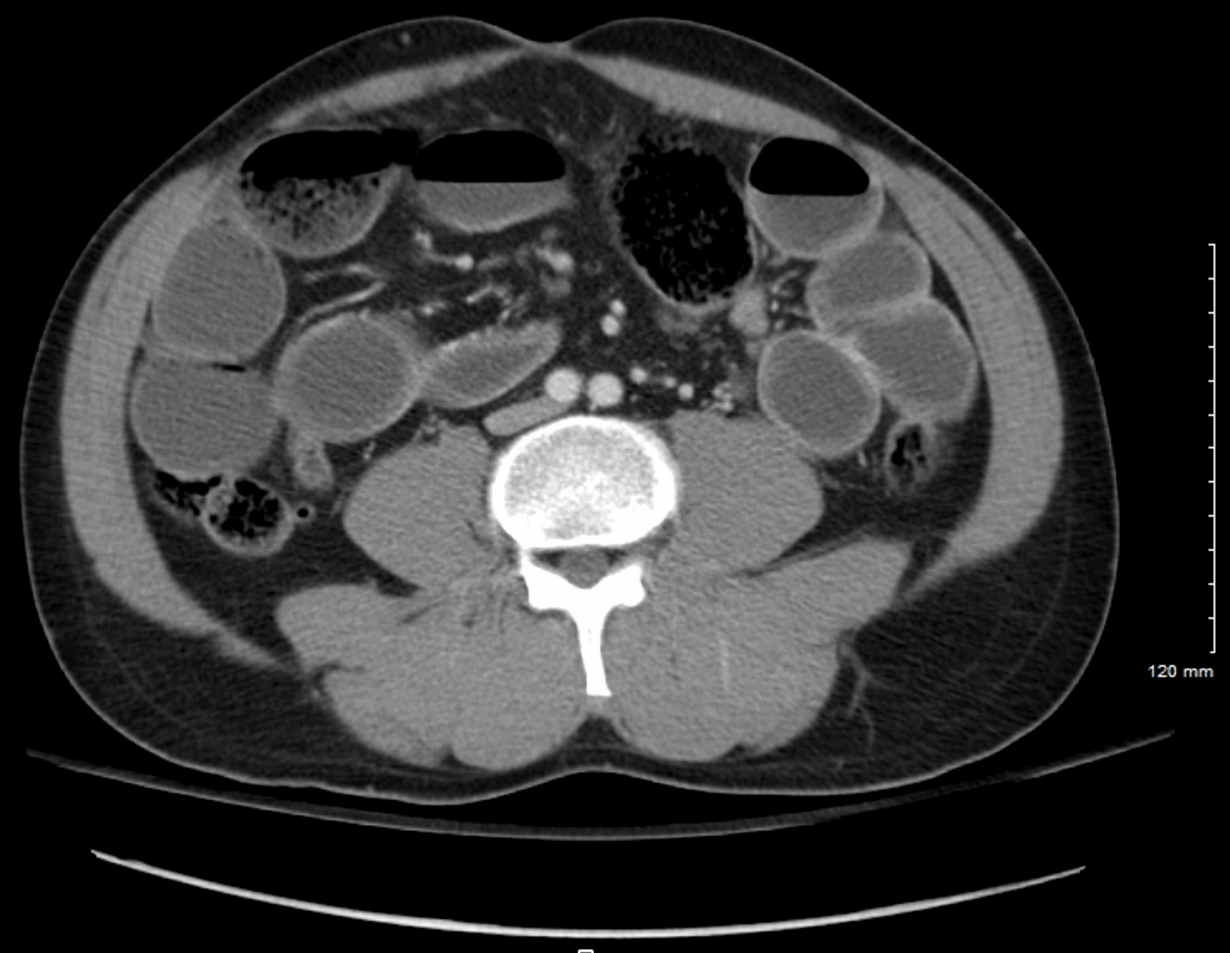
Case three: a CT scan of the abdomen and pelvis showing a small bowel obstruction, with the transition point in the right hemiabdomen at the site of a previous surgical change. There is a nonspecific bowel gas pattern, with no evidence of pneumoperitoneum. Moderate fecal loading is also present.

During the physical examination, the patient was afebrile and hemodynamically stable. His abdomen was mildly distended and tender, but no masses or organomegaly were detected. His extremities were normal, and his skin showed no rashes or lesions. Lab results showed a slightly elevated WBC count (15.1 × 109/L; normal range: 4.5 to 11.0 × 109/L) and elevated calcium levels (10.8 mg/dL; normal range: 8.6-10.3 mg/dL). His pulse rate was 77 beats per minute. Other laboratory parameters were within the normal range. At this point, the SBO was believed to result from adhesive disease stemming from previous surgical procedures. A CT scan (Figure 7) was ordered for further investigation, and immediate surgical intervention was deemed necessary as the patient did not respond to conservative therapy, including intravenous fluid and fluid exams.

**Figure 8:**
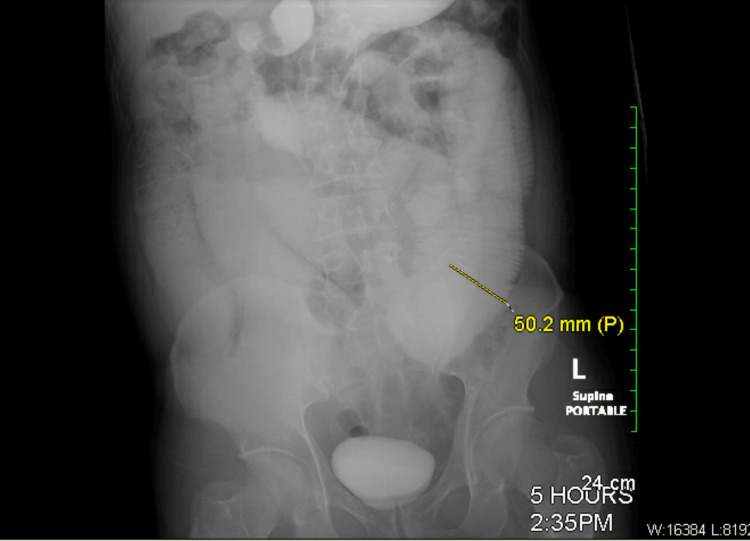
Case three: small bowel follow-through at a five-hour delay depicting small bowel obstruction

The SBFT study was conducted to analyze the extent of the obstruction further. In this process, 15 images were obtained from both pre-and post-oral contrast (Gastrografin) administration. As shown in Figure 8, five-hour delay exam findings indicated high-grade obstruction (yellow arrow) of the small bowel, which correlated with the CT results in Figure 7.

**Figure 9:**
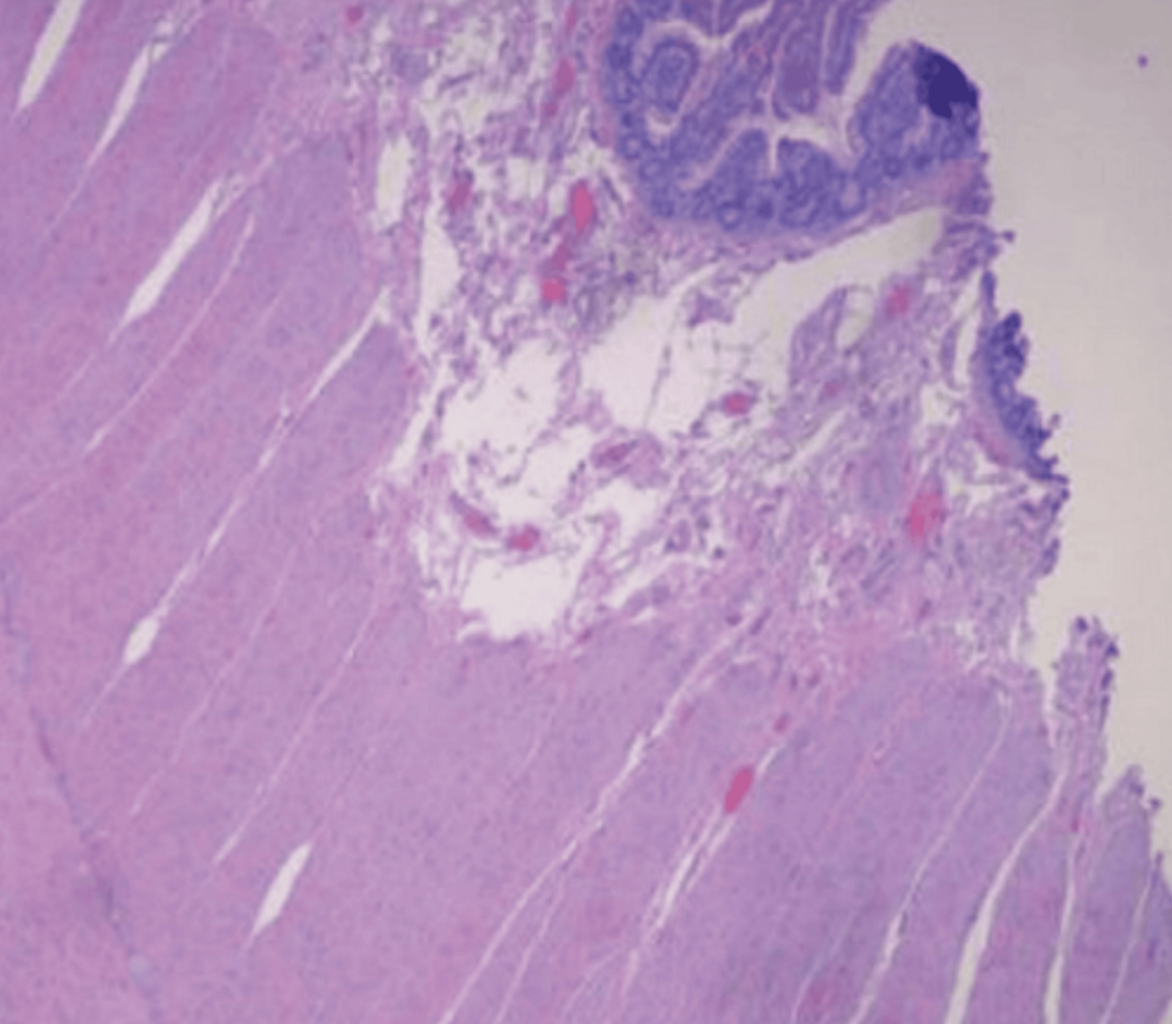
Case three: focal chronic inflammation, vascular congestion, and reactive changes seen on the bowel segment

The preoperative and postoperative diagnosis for the patient was a SBO, for which surgical intervention was opted. The procedure performed was an exploratory laparotomy with adhesiolysis, enteroenterostomy, and insertion of a Jackson-Pratt (JP) drain under general anesthesia. Histopathological findings of the resected bowel segment showed chronic inflammation, vascular congestion, focal hemorrhage on sectioning, and reactive changes seen on the bowel segment. Figure 9 shows histopathological findings.

Postoperative management protocol

A unique protocol was applied in the care of all three patients, including prompt diagnosis and intervention, which led to optimal outcomes despite underlying medical conditions and varying ages. Moreover, after recovery in the post-anesthesia care unit (PACU), all patients were transferred to the medical unit for interdisciplinary care. The patients also received daily wound care, pain management, multisystem observations, laboratory analysis, as well as respiratory, physical, occupational, and nutritional therapies. The use of incentive spirometers (I/S) and ambulation were encouraged and utilized throughout the patient's hospital stay. Upon completion of physical therapy, dietary consultations, mental evaluations, and post-discharge education, patients were discharged. In addition to discharge instructions, patients completed post-surgical follow-ups at the outpatient clinic, and no postoperative complications were reported. This comprehensive approach to the management of SBO allowed patients to receive optimal care and prevent postoperative complications and recurrences of the condition. 

## Discussion

Small bowel obstruction occurs when the small intestine becomes partially or completely obstructed. Various factors can lead to this condition, including adhesions, hernias, tumors, inflammatory bowel disease, and radiation therapy [1]. Adhesions are the most common cause of SBO and account for up to 74% of the cases [6]. They mostly occur after abdominal surgery or due to inflammation in the abdominal cavity [1]. Approximately 7% of the cases are attributed to Crohn’s disease, 5% to neoplasia, 2% to hernias, and 1% to radiation [6].

The diagnosis of SBO is made through a combination of physical examination, laboratory tests, and imaging studies, including CT scans and SBFT. Patients typically present with colicky abdominal pain, vomiting, and inability to pass flatus or stool if complete (no obstipation if partial) [7]. Physical examination is positive for hyperactive bowel sounds, which can later become absent, distended, and tympanitic abdomen [8]. Dilated loops of the bowel with air-fluid levels on plain film or CT scans are among the diagnostic tools available for SBO [9, 10].

**Figure 10:**
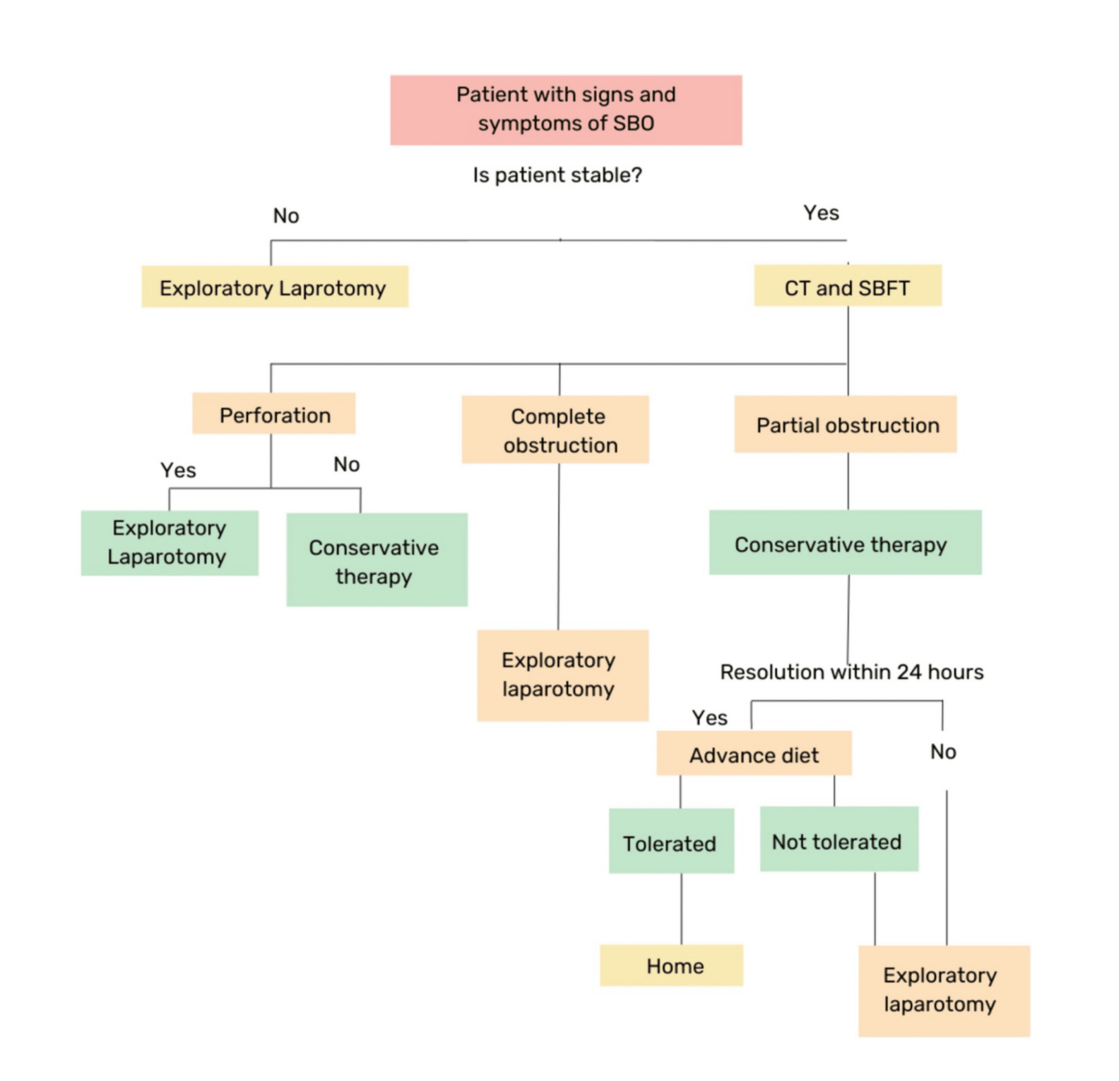
Flow chart illustrating the management approaches for small bowel obstructions SBO: small bowel obstruction; CT: computed tomography; SBFT: small bowel follow-through This figure has been created by the author, Abshiro H. Mayow, using data sourced from reference [11].

As illustrated in Figure 10, the approach to managing small bowel obstruction depends on factors such as the clinical presentation, type of obstruction, and findings from diagnostic imaging. The initial management of SBO involves bowel rest, nasogastric tube decompression, intravenous fluid resuscitation, and correction of electrolyte imbalances. The decision to perform surgery in patients with SBO depends on the cause of the obstruction, the severity of factors such as increased WBC, fever, and the presence of complications. Conservative treatment is administered in cases of partial obstruction without strangulation. In cases of complete obstruction, conservative therapy could be possible, but diagnostic methods such as CT or contrast studies are used to determine those who require early surgical intervention [11]. Patients with previous abdominal surgery, multiple episodes of SBO, or those with signs of bowel ischemia or perforation are more likely to require surgery [12]. Early surgical intervention prevents serious complications such as bowel necrosis, sepsis, and death [12].

Complications of SBO range from dehydration, electrolyte imbalances, bowel ischemia, and perforation, which can lead to sepsis and require emergency surgery [12-14]. Bowel ischemia was found to be the most common complication of SBO, occurring in 17% of cases [10]. Advanced age and bowel ischemia increase the mortality rate in patients with SBO to 8.4% [15]. This leads to tissue damage and potentially perforation, which can cause sepsis and require emergency surgery. Dehydration, which occurs due to vomiting and reduced oral intake, leads to electrolyte imbalance [10]. This can cause complications such as cardiac arrhythmias, seizures, and coma [10]. Oral water-soluble contrast agents decrease the risk of dehydration and electrolyte imbalance [9]. Prompt diagnosis and appropriate management are crucial to minimize the risk of complications and ensure a good prognosis [11].

This case series describes three patients, each with different etiologies and presenting with complex small bowel obstruction. Such a comprehensive approach to treating SBO allowed patients to receive optimal and continuous care to prevent post-surgical complications and SBO recurrence. The findings in this study corroborate and advance the current therapy and management of small bowel obstruction regardless of a patient's existing medical conditions, age, and factors that might precipitate the problem.

## Conclusions

Prompt diagnosis and surgical intervention can significantly improve outcomes in individuals with a small intestinal blockage. This case series highlighted the management of three patients who required surgical intervention for SBO due to complete obstruction or associated complications. The advent of CT scans in aiding precise diagnosis avoids delays and enables physicians to make appropriate decisions in patient care. According to the guidelines and literature available, some patients can be managed conservatively depending on the severity of symptoms or etiology, but the decision for surgical intervention should be made swiftly once indications are present to avoid intestinal necrosis, sepsis, or even death. A high index of suspicion is advised for patients with extensive surgical histories who present with abdominal pain, constipation, or vomiting. Comprehensive postoperative care and adequate follow-up are critical in avoiding surgical complications and recurrence. However, this study has some limitations, including a small sample size, which limits its validity. We recommend a multidisciplinary approach based on a patient's specific presentation and medical history and developing individualized treatment plans prior to generalizing our protocol to all patients presenting with SBO.
